# How efficient are referral hospitals in Uganda? A data envelopment analysis and tobit regression approach

**DOI:** 10.1186/s12913-016-1472-9

**Published:** 2016-07-08

**Authors:** Paschal N. Mujasi, Eyob Z. Asbu, Jaume Puig-Junoy

**Affiliations:** Department of Health Studies, Health Economist; Doctoral student, University of South Africa, College of Human Sciences, 1 Preller Street, Muckleneuk, Pretoria, 0002 South Africa; Division of Health System Financing, Health Authority, Abu Dhabi, United Arab Emirates; Department of Economics and Business; and Centre for Research in Health and Economics (CRES), Universitat Pompeu Fabra, Ramón Trias Fargas 25-27, 08005 Barcelona, Spain

**Keywords:** Data envelopment analysis, Hospital efficiency, Technical efficiency, Tobit model, Uganda

## Abstract

**Background:**

Hospitals represent a significant proportion of health expenditures in Uganda, accounting for about 26 % of total health expenditure. Improving the technical efficiency of hospitals in Uganda can result in large savings which can be devoted to expand access to services and improve quality of care. This paper explores the technical efficiency of referral hospitals in Uganda during the 2012/2013 financial year.

**Methods:**

This was a cross sectional study using secondary data. Input and output data were obtained from the Uganda Ministry of Health annual health sector performance report for the period July 1, 2012 to June 30, 2013 for the 14 public sector regional referral and 4 large private not for profit hospitals. We assumed an output-oriented model with Variable Returns to Scale to estimate the efficiency score for each hospital using Data Envelopment Analysis (DEA) with STATA13. Using a Tobit model DEA, efficiency scores were regressed against selected institutional and contextual/environmental factors to estimate their impacts on efficiency.

**Results:**

The average variable returns to scale (Pure) technical efficiency score was 91.4 % and the average scale efficiency score was 87.1 % while the average constant returns to scale technical efficiency score was 79.4 %. Technically inefficient hospitals could have become more efficient by increasing the outpatient department visits by 45,943; and inpatient days by 31,425 without changing the total number of inputs. Alternatively, they would achieve efficiency by for example transferring the excess 216 medical staff and 454 beds to other levels of the health system without changing the total number of outputs. Tobit regression indicates that significant factors in explaining hospital efficiency are: hospital size (*p* < 0.01); bed occupancy rate (*p* < 0.01) and outpatient visits as a proportion of inpatient days (*p* < 0.05).

**Conclusions:**

Hospitals identified at the high and low extremes of efficiency should be investigated further to determine how and why production processes are operating differently at these hospitals. As policy makers gain insight into mechanisms promoting hospital services utilization in hospitals with high efficiency they can develop context-appropriate strategies for supporting hospitals with low efficiency to improve their service and thereby better address unmet needs for hospital services in Uganda.

## Background

Besides equity and financial protection, the pursuit of efficiency is a key policy objective of policy makers in most health systems [1]. This is much more evident in Africa where the ability to adequately meet health care needs is exacerbated by extensive inefficiencies, especially within the hospital sector [2–8]. Conservative estimates indicate that globally, about US$ 300 billion is lost annually to hospital-related inefficiency [9].

Hospitals represent a significant proportion of health expenditures. In Uganda for example, about 26 % of Total Health Expenditure (THE) is through hospitals [10]. Hospital expenditures accounted for 37 % of Government Current Health Expenditure (GCHE) in 2011/12. Of this, 23 % was spent on Regional referral hospitals, 66 % in primary health care hospitals and 11 % in specialty hospitals/Institutions [11]. A health system’s efficiency is thus to a great extent determined by the efficiency of its hospitals. For example, a modeling exercise in Australia demonstrated that a 4 % gain in the efficiency of hospitals would contribute to a 1.9 % increase in the overall efficiency of the country’s health system signifying the important role played by hospitals in influencing the overall health systems efficiency [12]. Thus improvement in the pure technical and scale efficiency of hospitals may result in large savings in healthcare expenditures, which could be devoted to expand access to preventive, promotive, curative and rehabilitative services and improve quality of care. This will contribute significantly to a country’s endeavors towards achieving universal health coverage in line with the Sustainable Development Goals.

### Study context

Located in East Africa, with an estimated population of 34.856 M in 2014 [13], Uganda is a low income country served by a healthcare delivery system that comprises of the public sector, private sector and the non-governmental organizations (NGO)/private not profit sector. As of 2010, the health care delivery system comprised of 129 hospitals, 177 Health Centre IVs, 1082 health center IIIs and 3006 Health Centre IIs [14]. At the apex of the healthcare delivery system are national referral hospitals below which are regional referral hospitals to which district hospitals refer. There is a district health care delivery system below the district hospitals comprising health center IVs, IIIs and health center IIs, and village health teams. The district hospitals act as referral centers for the district health care delivery system. The regional referral hospitals act as referral centers for several districts within their catchment area [15].

The Uganda National Health Policy indicates that efficiency is currently not well addressed in the way resources are mobilized, allocated and used [16]. Thus, one of the Uganda Ministry of Health objectives in the Health sector strategy and investment plan (HSSIP 2010/11-2014/15) is to improve the efficiency and effectiveness of health services [14]). For this to be achieved, information on the current level of efficiency in delivery of the various health services and the drivers of inefficiency will be required.

The Uganda Ministry of Health (MOH) routinely analyses and reports on efficiency of regional referral and other hospitals in its annual health sector performance reports. However, the analysis explores efficiency in a general sense using ratio indicators, mainly, the standard unit of output (SOUs) per health worker. The analysis does not take into account other factors of production (e.g. Beds, finances etc.) used by the hospitals in the production of the outputs. Additionally, the report does not include any deep analysis of the factors affecting hospital efficiency and the required changes to improve efficiency. Such information would be useful to the MOH in taking the required actions to improve efficiency, which is one of the health policy objectives. Additionally, there are no recently published facility efficiency studies in Uganda, with the available studies having been published in 2010 and 2008 [17, 18] using 1999-2003 data. These studies can be considered outdated given that a lot has happened since then in terms of the country’s socioeconomic and health development.

This study tries to address these gaps. The study demonstrates how a study of hospital efficiency using Data Envelopment Analysis (DEA) and routinely reported data can inform decision-making.

### Conceptual framework

#### Hospitals as production units

Hospitals use multiple health system inputs to produce multiple health service outputs through a production process. Inputs (labor and capital) combine via medical and surgical care to produce outputs. While the ultimate output of healthcare is the marginal change in health status, this is difficult to measure in most data sets, and so intermediate outputs – episodes of care (e.g. number of operations and outpatient visits) – usually become the primary study outputs. This production process does not occur in a vacuum; it can be influenced by a number of environmental factors both internal and external to the hospital which may influence how efficiently the production process occurs [19]. Often these factors are considered to be uncontrollable by the hospital managers. The factors are theorized either to affect the production process itself or to influence directly the efficiency of the process [20].

**Fig. 1:**
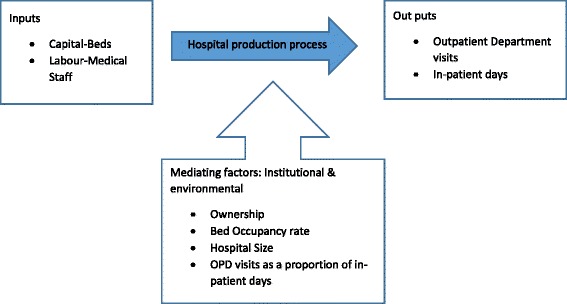
Uganda referral hospital inputs, process, out puts and influencing factors

Figure 1 depicts the relationship between health system inputs, the production process, and the outputs/results. This forms the framework for our study.

#### Efficiency concepts

Skaggs and Carlson [21] define economic efficiency as obtaining the maximum benefit from a given cost or minimizing the cost of a given benefit. Economic efficiency comprises both technical efficiency (producing without waste) and allocative efficiency (allocating resources to their most high value uses).

Technical efficiency is achieved when a health decision making unit (DMU) produces a given level of health service outputs with the least health system inputs, e.g. health workforce, pharmaceutical and non-pharmaceutical supplies and capital inputs (buildings, beds, equipment, vehicles etc.).

The technical efficiency of a health DMU is decomposed into pure technical efficiency and scale efficiency. Pure technical efficiency denotes technical efficiency that cannot be attributed to deviations from optimal scale (scale efficiency). Scale efficiency on the other hand is a measure of the extent to which a health decision making unit deviates from optimal scale (defined as the region in which there are constant returns to scale in the relationship between outputs and inputs) [22, 23].

Salvatore [24] defines returns to scale as the extent to which health system output changes as a result of a change in the quantity of all health system inputs used in production. A constant return to scale is achieved when the quantity of health service outputs increase in the same proportion as the increase in the quantity of all inputs. An increasing return to scale is achieved if output increases by a greater proportion than the increase in inputs and a decreasing return to scale is achieved where output increases by a smaller proportion than the increase in inputs.

In the context of health, allocative efficiency describes the use of health system inputs by a health facility or decision-making unit (DMU) in the proportion that minimizes the cost of production, given input prices [22–24]. On the other hand, technical efficiency describes the production by a health DMU of the optimal/maximum quantity of outputs from the available health system inputs [22–24].

Efficiency is never absolute; instead it is always assessed relative to some criterion. Cooper et al. [25] explain that a DMU is to be rated as fully (100 %) efficient on the basis of available evidence if and only if the performances of other DMUs does not show that some of its inputs or outputs can be improved without worsening some of its other inputs or outputs. Efficiency scores are bounded by zero (totally inefficient) and one (totally relatively efficient).

#### Data envelopment analysis

DEA is a non-parametric, data driven approach that uses linear programming techniques to compute the efficiency scores for each DMU in a data set. DMUs that are technically efficient have a score of 1 or 100 %, whereas inefficient ones have efficiency scores of less than 1 (i.e. less than 100 %).

In DEA the efficiency of a DMU (referral hospitals in this case) is measured relative to a group’s observed best practice. This implies that the benchmark against which to compare the efficiency of a particular referral hospital is determined by the group of referral hospitals in the study and not a value fixed by hospitals outside of the group.

DEA easily accommodates multiple inputs and outputs without the requirement for a common denominator of measurement. This makes it particularly suitable for analyzing the efficiency of hospitals as they use multiple inputs to produce many outputs. Furthermore, it provides specific input and output targets that would make an inefficient hospital relatively efficient. It also identifies efficient peers for those hospitals that are not efficient. This helps the inefficient hospitals to emulate the functional organization of their peers so as to improve their efficiency.

However, like many other empirical methods, DEA has its limitations. First, it produces results that are sensitive to measurement error. For example, if one hospital’s inputs are understated or its outputs overstated, it can become an outlier and significantly reduce the efficiency of other hospitals. Second, DEA measures efficiency relative to the best practice within hospitals in the particular sample.

A growing number of African countries have undertaken health facility efficiency studies using data envelopment analysis (DEA) to guide the development of interventions to reduce waste of scarce health system resources. Since 2000 studies have been undertaken in a number of countries, including Angola [2], Namibia [3], Botswana [4], Zambia [5] Kenya [6, 26], Benin [27], Burkina Faso [28], Ethiopia [29], Ghana [30–32], Nigeria [33], Seychelles [34], Sierra Leone [35, 36], South Africa [7, 37], Uganda [18] and Eritrea [38]. These studies demonstrate that DEA is an important tool for policy advice.

### Research questions and objectives

We address three research questions: What was the technical efficiency of regional referral and large private not for profit (PNFP) hospitals in Uganda in FY 2012/2013? What is the scope for increase in outputs of the same quality by the regional referral hospitals without increasing the amount of resources used? How do institutional and contextual/environmental variables affect efficiency of regional referral hospitals in Uganda?

The specific objectives of our study are: (a) to estimate the technical and scale efficiency of regional referral hospitals in Uganda in FY 2012/2013 (b) to estimate the magnitudes of output increases of the same quality that would have been required to make relatively inefficient hospitals more efficient; and (c) to determine the impact of institutional and/or contextual/environmental variables on hospital inefficiencies. We use two-stage DEA: (i) to estimate efficiency of the regional referral hospitals; and (ii) explain the inefficiencies using Tobit regression.

## Methods

### Study design

This was a cross sectional study using secondary data.

### Study population

In Uganda, regional referral hospitals offer specialist clinical services such as psychiatry, Ear, Nose and Throat (ENT), ophthalmology, higher level surgical and medical services, and ancillary services (laboratory, medical imaging and pathology). They are also involved in teaching and research. This is in addition to the following services provided by general hospitals: preventive, promotive, curative, maternity, in-patient health services, surgery, blood transfusion, laboratory and medical imaging services [14].

For the financial year 2012/13, there were a total of 14 public sector regional referral hospitals operational in Uganda. Additionally, there were 4 large PNFP hospitals with the same range of services as public sector regional referral hospitals [39]. Thus, our study population comprised of 18 hospitals.

A data base of inputs and outputs for all the 14 public sector regional referral and the 4 PNFP hospitals was compiled from various sections of the 2012/2013 MOH annual health sector performance report [39]. Our final study sample comprised of 17 hospitals. One PNFP hospital was excluded from the study for lack of data.

### Data and variables

Our study uses Uganda hospital data for the 2012/2013 Financial year (July 1, 2012 to June 30, 2013) as reported by the MOH in the annual health sector performance report (AHSPR) to explore the technical efficiency of regional referral and large private not for profit (PNFP) hospitals during that period.

#### Input and output variables

Data was assembled for 3 different inputs (medical staff, hospital beds, budget) and 5 outputs (OPD visits, in-patient days, deliveries, major operations and immunizations). Based on completeness of available data, final selection was limited to 2 input and 2 outputs. The inputs included total number of medical staff and hospital beds. The outputs included outpatient department (OPD) visits and in-patient days. We presume these capture most of the hospital activities. For example surgeries are also subsumed in inpatient and outpatient care (if day case surgeries).

The input and output variables are described in Table 1.

**Table 1:** Definition of study variables

Variables	Definition	Measurement	Data Source
Input variables			
BEDS	Beds	Total number of hospital beds during the financial year	Annual Health Sector Performance Report 2012/2013
STAFF	Medical Staff	Total number of doctors, nurses, Clinical officers, Laboratory technicians and anesthetic officers.	Annual Health Sector Performance Report 2012/2013
Output variables			
INPATDAYS	In Patient days	Total number of inpatient days for the Financial year	Annual Health Sector Performance Report 2012/2013
OPD	Outpatient visits	Total number of outpatient visits during the Financial year	Annual Health Sector Performance Report 2012/2013
Explanatory variables			
BOR	Bed Occupancy Rate	Proportion of beds which are occupied over a specified period of time	Annual Health Sector performance report 2012/2013
OPDIPD	Outpatient visits as a proportion of inpatient days;	Total Outpatient department visits divided by the total inpatient days	Annual Health Sector performance report 2012/2013 (calculated)
ALOS	Average Length of Stay	Average number of days that patients spend in hospital. Measured by dividing the total number of days stayed by all inpatients during a year by the number of admissions	Annual Health Sector performance report 2012/2013
SIZE	Hospital Size	Number of beds in the hospital. 1 if >190 and 0 if <190	Annual Health Sector performance report 2012/2013
OWN	Ownership	Governing authority of hospital. 1 if Government/private hospital owned; 0 if NGO/PNFP	Annual Health Sector performance report 2012/2013
TEACH	Teaching status	Whether a hospital is attached to a university or not for the purpose of training medical students (doctors & Pharmacists)	Individual hospital website
DIST	Distance	Distance of the teaching hospital from the capital city. 1 if and 0 if	Individual hospital website
POP	Population size	Total population of districts in the hospital’s catchment area.: 1 if and 0 if	Uganda National Population and Housing Census 2014 (calculated)

The choice of the above-mentioned inputs and outputs was guided by three considerations, namely: past studies undertaken of hospitals in Africa, which also employed similar inputs and outputs [2-8;28-38;]; the availability of relevant data in the ministry of health annual health sector performance report for FY 2012/13 [39]; and the availability of data that is routinely compiled by hospitals in order to demonstrate ways in which the Uganda MOH can get added informational value from such data without investing extra resources. The inputs and outputs data were used as reported in the ministry’s annual health sector performance report without any processing or manipulation.

#### Explanatory variables

The literature indicates that some of the factors that impact health facility efficiency include, catchment population, distance, location (urban/rural), ownership (profit/not-for-profit), teaching status, payment source (out-of-pocket/health insurance), occupancy rate, average length of stay, outpatient visits as a proportion of inpatient days, and quality [7, 40, 41]. These can be considered as explanatory variables for hospital efficiency.

In our study, we selected the explanatory variables based on availability of data. They are also described in Table 1.

#### Data management and analysis methods

The data collected on inputs, outputs and explanatory variables were entered into a computer using Excel software. The excel data were then exported into STATA 13. Data analysis was conducted in two stages. STATA 13 was used for data analysis in both stages.

### First stage analysis-data envelopment analysis (DEA)

We used DEA to calculate the efficiency scores for each of the hospitals in the sample. Using STATA, we run an output-oriented model with Variable Returns to Scale (VRS) to estimate the individual hospital efficiency scores. The VRS model estimated the pure technical efficiency and scale efficiency for each of the sample hospitals. From the VRS model, we analyzed whether a hospital’s production indicated increasing return to scale, constant return to scale, or decreasing return to scale.

The VRS model was adopted under the assumption that in practice there are variable returns to scale and not all hospitals are operating at an optimal scale. Also, given the existence of unmet need and low quality of care, we wanted to investigate the potential efficiency savings that can be used to expand care and/or improve quality.

According to Coelli [42], where DMUs are given a fixed quantity of resources (inputs) and asked to produce as much output as possible, an output orientation is more appropriate. In the Ugandan context hospitals have a more or less fixed quantity of inputs and managers are expected to produce as much output as possible. For example, the staffing capacity of each hospital is determined centrally by the MOH or hospital managing authority, and thus individual hospital managers do not have any control over the size of the health workforce. Even when inputs such as beds and staff are underutilized, it is not within the managers’ power to dispose of them.

### Model specification

Assuming that there are j referral hospitals, each with n hospital inputs and m hospital outputs, the relative efficiency score of a given hospital (θ_0_) is obtained by solving the following output-orientated CCR DEA linear programming model:

$$ \mathrm{Maximise}\kern0.5em \theta \kern0.5em =\kern0.5em {U}_1{Y}_{1_o}\kern0.5em +\kern0.5em {U}_2{Y}_{2_o}\kern0.5em +\kern0.5em \cdots +\kern0.5em {U}_r{Y}_{r_o}\kern0.5em \Big(=\kern0.5em {\displaystyle \sum_{r\kern0.5em =\kern0.5em 1}^s{U}_r{Y}_{r_o}\Big)} $$

Subject to the constraints that:

$$ {v}_1{x}_{1_o}\kern0.5em +\kern1em {v}_2{x}_{2_o}\kern0.5em +\cdots +\kern0.5em {v}_m{x}_{m_o}\kern0.5em =\kern0.5em {\displaystyle \sum_{i\kern0.5em =\kern0.5em 1}^m{v}_i{x}_{i_o}\kern0.5em =\kern0.5em 1} $$

$$ {x}_{1j}\kern0.5em +\kern0.5em {v}_2{x}_{2j}\kern0.5em +\cdots +\kern0.5em {v}_m{x}_{mj} $$

Where:

θ_0_ = the efficiency score of hospital 0;

X_nj_ = the amount of health system input n utilized by the j^th^ hospital;

Y_mj_ = the amount of health system output m produced by the j^th^ hospital;

u_m_ = weight given to health system output m;

v_n_ = weight given to output n

One of the main drawbacks of DEA is that the resulting efficiency scores are sensitive to the presence of DMUs that perform extremely well (outliers), either due to outstanding practice or errors in the data. In either case, the results for the remaining DMUs become shifted towards lower efficiency levels, the efficiency frequency distribution becomes highly asymmetric, and the overall efficiency scale becomes nonlinear. Thus, we used jackknife analysis to test for the robustness of the DEA technical efficiency measures and assess if there were extreme outliers affecting the frontier and efficiency scores. In conducting the jackknife analysis, we dropped each efficient hospital one at a time from the analysis and efficiency scores re-estimated. We tested the similarity of the efficiency rankings between the model with all the hospitals included and those based on dropping each efficient hospital one at a time using the Spearman rank correlation coefficient. The efficiency scores obtained were robust as indicated by Spearman rank correlation coefficient, which was very close to one.

### Second stage- econometric analysis (Tobit Regression)

In the second stage, the DEA efficiency scores computed in the previous section were regressed against some institutional factors which are at the discretion of the hospital management and selected contextual/environmental (non-discretionary) factors that are beyond their control to estimate their impacts on efficiency. These factors are already described in Table 1.

Thus, using the VRS technical efficiency scores as a dependent variable and given that the scores are right-censored (i.e. upper limit of 100%), a Tobit regression model was used to estimate the adjusted efficiency scores for each hospital.

The Tobit obtains estimates of the linear Tobit model, where the dependent variable is either zero or positive. The method used was maximum likelihood under the assumption of homoscedastic normal disturbances.

The following Tobit regression Model was used:

$$ \mathrm{Tobit}\kern0.5em \left({Y}_i\right)\kern0.5em =\kern0.5em {a}_0\kern0.5em +\kern0.5em {a}_1{x}_{j_1}\kern0.5em +\kern0.5em {a}_2{x}_{j_2}\kern0.5em +\kern0.5em {a}_3{x}_{j_3}\kern0.5em +\cdots +\kern0.5em {\varepsilon}_j $$

Where:

yj is the variable return to scale efficiency score for the jth hospital,

xj are the explanatory variables,

εj are the disturbance term assumed to be normally distributed with mean μ and standard deviation σ

α are the Tobit coefficients which indicate how a one unit change in an independent variable x_i_ alters the latent dependent variable y_i_. Sometimes the values of the tobit coefficients cannot be interpreted but their signs are helpful for interpreting the results of study

Following Asbu [43], the VRS DEA technical efficiency scores were transformed into inefficiency scores, left-censored at zero using the formula:

$$ \mathrm{Inefficiency}\kern0.5em \mathrm{Score}\kern0.5em =\kern0.5em \left(\frac{1}{DEATE\kern0.5em  score}\right)\kern0.5em -\kern0.5em 1 $$

The initially estimated general model contained all the identified explanatory variables and was:

$$ \mathrm{Ineff} = \upalpha + {\upbeta}_1\mathrm{OPDIPD} + {\upbeta}_2\mathrm{B}\mathrm{O}\mathrm{R} + {\upbeta}_3\mathrm{SIZE} + {\upbeta}_4\mathrm{OWN} + {\upbeta}_5\mathrm{ALOS} + {\upbeta}_6\mathrm{TEACH} + {\upbeta}_7\mathrm{DIST} + {\upbeta}_8\mathrm{POP} + {\upvarepsilon}_{\mathrm{i}} $$

Where β is the vector of unknown parameters or coefficients; and ε i is the stochastic/random error term. We estimated the Tobit regression using Stata 13 for Windows. By estimating the empirical model, we wished to test two hypotheses: First, in order to test the overall significance of the model, we state the joint null hypothesis as H0: β1 = β2 = β3 = β4 = β5 = 0 and the alternative hypothesis HA : β1 = β2 = β3 = β4 = β5 = β6 = β7 = β8 ≠ 0. The joint null hypothesis is tested using the likelihood ratio test (LL).

Second, we wished to test the hypothesis that βn is not significantly different from zero in either direction. Thus, the null (H0) and alternative hypotheses (HA) are: H0: βn = 0 ; and HA : βn ≠ 0 The individual null hypotheses are tested using the t-distribution test.

However, the objective was to estimate a parsimonious tobit model that would help explain the observed inefficiencies. Such a model would be significant based on the Chi square. Thus, through an iterative process, we run several models containing various combinations of the explanatory variables.

The finally selected empirical model based on the Chi Square was:

$$ \mathrm{Ineff} = \upalpha +{\upbeta}_1\mathrm{OPDIPD}+{\upbeta}_2\mathrm{B}\mathrm{O}\mathrm{R}+{\upbeta}_3\mathrm{SIZE} + {\upbeta}_4\mathrm{OWN} + {\upvarepsilon}_{\mathrm{i}} $$

Based on past two-stage hospital efficiency studies [10], we would expect a negative relationship between the Ineff and OPDIPD, and thus, β1 should a priori assume a negative sign. We would expect a negative relationship between the Ineff and OPDIPD, and thus, β1 should a priori assume a negative sign. Tobit coefficients indicate how a one unit change in an independent variable x_i_ alters the latent dependent variable y*.

### Ethical clearance

This study is entirely an analysis of data from published secondary sources. Since human subjects were not involved, it did not require ethical clearance.

## Results

### Descriptive analysis of inputs and output variables

Table 2 presents the descriptive statistics (sum, minimum, maximum, mean and standard deviation) for inputs and outputs of referral hospitals in our sample (public sector regional referral and large Private not for Profit) during the FY 2012/13 (July 1, 2012 to June 1, 2013). During the study period, the 17 hospitals received 2,442,117 outpatient department visits and provided 1,484,853 inpatient days of care. These outputs were produced using a total of 3433 Medical staff and 4874 hospital beds.

**Table 2:** Descriptive statistics of the input, output and explanatory variables for Regional Referral and large PNFP hospitals (*n* = 17)

	Mean	Standard deviation	Minimum	Maximum
Inputs				
Number of Clinical staff	202	88	97	453
Number of beds	287	117	100	482
Outputs				
Number of outpatient department visits	143,654	51,339	35,390	209,032
Patient days	87,344	41,052	24,382	176,671

There was wide variation in both output and inputs across the different hospitals. The outpatient department visits varied from a minimum of 35,390 to a maximum of 209,032. The inpatient days of care provided varied from a minimum of 24,382 days to 176,671 days. In terms of inputs there was considerable variation as well: the number of medical staff varied between 97 and 453; and hospital beds varied between 100 and 482.

### Descriptive analysis of explanatory variables

Table 3, shows the distribution of explanatory variables in the study sample.

**Table 3:** Distribution of explanatory variables

	Mean	Standard deviation	Minimum	Maximum
Continuous variables				
Bed Occupancy Rate (BOR)	81.4 %	19.9 %	40.8 %	100 %
Outpatient department visits as a proportion of In-patient stays (OPDIPD)	2	1.27	0.48	5.30
Average Length of Stay (ALOS)-days	4.6	1.2	3	7
		Description	Number	Percentage
Categorical Variables				
Catchment Population (POP)- 1 if catchment population less than 1.5 million and 0 if greater than 1.5 million		0	8	47 %
		1	9	53 %
Distance from city (DIST) 1 if hospital located greater than 200Km Kilometres from capital city and 0 if located less than 200 km		1	14	82 %
		0	3	18 %
Teaching Status (TEACH) 1 if Teaching Hospital and 0 if not		0	15	88 %
		1	2	12 %
Hospital Ownership (OWN) -1 if government/MOH and 0 if NGO/PNFP		0	3	18 %
		1	14	82 %
Hospital Size (SIZE)-1 if >190 hospital beds and 0 if <190 hospital beds		0	4	24 %
		1	13	76 %

The majority of hospitals in the sample (82 %) were public hospitals owned by the government. Two hospitals (12 %) were teaching hospitals. Nine hospitals (53 %) had a catchment population greater than 1.5 million. Three hospitals (18 %) were located within 200 kilometers from the capital city.

For purposes of this study, hospitals with more than 190 hospital beds (lower quantile) were categorized as big hospitals. Based on this categorization, 24 % of the hospitals in our study sample could be considered small hospitals with the remaining 76 % categorized as large hospitals.

The bed occupancy rate varied from 40.8 to 100 % with a mean of 81.4 % and standard deviation 19.9 %. The proportion of outpatient department visits as a proportion of inpatient days varied from 0.44 to 5.30 with a mean of 2 and standard deviation of 1.27. The average length of stay was 4.6 days with a standard deviation of 1.2 days. The average length of stay ranged from 3 to 7 days.

### Technical efficiency

Table 4 shows the individual hospital DEA scores for constant returns to scale technical efficiency, variable returns to scale technical efficiency (pure technical efficiency), scale efficiency, and returns to scale. The table also shows the efficiency reference set for each hospital, which refers to the group of hospitals against which DEA located the relatively inefficient hospitals and the magnitudes of inefficiency.

**Table 4:** Output oriented DEA efficiency scores for Table 2 descriptive statistics of the input and outputs for Regional Referral and large PNFP hospitals (*n* = 17)

Hospital	Efficiency scores			Returns to scale	Reference set (lambda weights)
	CRS_TE	VRS_TE	Scale		
Gulu	1	1	1	Constant returns to scale	
Hoima	1	1	1	Constant returns to scale	
Mbale	0.940133	1	0.940133	Diminishing returns to scale	
Moroto	1	1	1	Constant returns to scale	
Masaka	0.8954	1	0.8954	Diminishing returns to scale	
Lira	0.900694	1	0.900694	Diminishing returns to scale	
Nsambya	0.431078	1	0.431078	Diminishing returns to scale	
St. Mary’s Lacor	0.692294	1	0.692294	Diminishing returns to scale	
Mbarara	0.861657	0.976592	0.88231	Diminishing returns to scale	Masaka (0.406617), Mbale (0.309851), Gulu (0.097515), Hoima (0.162609)
Soroti	0.850387	0.941373	0.903348	Diminishing returns to scale	Gulu (0.260962) Hoima (0.250726) Mbale (0.323689); Moroto (0.105996)
Fort Portal	0.76477	0.921675	0.829762	Diminishing returns to scale	Hoima (0.037365) Masaka (0.884309)
Jinja	0.70878	0.86767	0.816877	Diminishing returns to scale	Masaka (0.640645) St. Mary’s Lacor (0.114007); Mbale (0.113018)
Arua	0.75922	0.855481	0.887478	Diminishing returns to scale	Gulu (0.055922) ;Mbale (0.008273), Masaka (0.791286)
Mubende	0.847976	0.850297	0.99727	Increasing returns to scale	Gulu (0.064533), Hoima (0.318159); Mbale (0.005493); Moroto (0.462112)
Kabale	0.81319	0.843276	0.964323	Increasing returns to scale	Masaka (0.125589) Hoima (0.702674); Lira (0.015013)
Rubaga	0.524107	0.762422	0.687424	Increasing returns to scale	Gulu (0.126505) Nsambya (0.19374);Masaka (0.442177)
Naguru	0.509215	0.523887	0.971994	Increasing returns to scale	Gulu (0.408324), Moroto (0.077042)
Min	0.431078	0.523887	0.431078		
Max	1	1	1		
Mean	0.794053	0.914275	0.870611		
SD	0.174409	0.125919	0.149222		

Overall, 3 hospitals (18 %) were operating under constant returns to scale, implying that they were efficient (both pure technical and scale efficiency). A percentage change in inputs is accompanied by the same percentage change in outputs.

Ten hospitals (59 %) were operating under diminishing returns to scale, implying that their health service outputs would increase by a smaller proportion compared to any increase in health service inputs. These hospitals would have to reduce their size to achieve optimal scale.

Four hospitals (24 %) were operating under increasing returns to scale implying that their health service outputs would increase by a greater proportion compared to any increase in health services inputs. These hospitals would need to increase their size to achieve optimal scale, i.e. the scale at which there are constant returns to scale in the relationship between inputs and outputs.

**Fig. 2:**
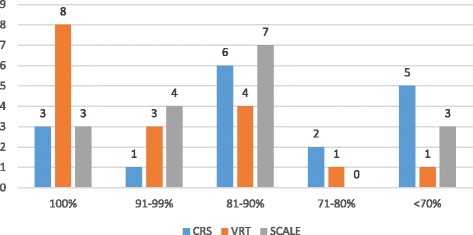
Distribution of Efficiency scores

Figure 2 shows the distribution of pure technical, constant returns to scale, and scale efficiency scores among the hospitals in the sample.

The figure demonstrates that constant return to scale inefficiency was a widespread problem in our sample, with only three hospitals (18 %) being CRS technically efficient. The mean constant return to scale technical efficiency was 79.4 %, with a standard deviation of 17.4 %. The constant return to scale technical efficiency score varied from a minimum of 43.1 % to a maximum of 100 %.

Scale inefficiency was equally a widespread problem in our sample, with only three hospitals (18 %) being Scale technically efficient. The average scale efficiency score was 87.1 % (standard deviation = 14.9 %), meaning that on average, the scale inefficient hospitals could theoretically reduce their size by 12.9 % without affecting their current output levels.

Pure technical inefficiency was less of a problem, with slightly less than half (Eight hospitals-47 %) of the hospitals being VRS technically efficient. The overall sample average pure technical efficiency score was 91.4 % (standard deviation = 12.6 %), meaning that inefficient hospitals could, on average, produce 8.6 % more health service outputs using their current inputs. The lowest pure technical efficiency score was 52.4 %.

### Pearson correlation analysis

Before conducting the tobit regression analysis, we analyzed the correlation between the traditional partial indicators of efficiency that were included in the explanatory variables list in Table 1 with different efficiency measures obtained using DEA. The indicators considered were Bed occupancy Rate (BOR), Average Length of Stay (ALOS) and proportion of outpatient department visits as a proportion of inpatient days (OPDPID). Ideally, these indicators should not be considered as explaining factors of efficiency scores especially if they are highly correlated to the efficiency scores, because they only partially measure the relation between some inputs and outputs.

The average length of stay (ALOS) and the proportion of outpatient department visits as a proportion of inpatient days were not significantly correlated with any of the efficiency scores. The bed occupancy rate however was positively and highly correlated with the constant returns to scale and scale efficiency scores but not the technical efficiency score. The higher the bed occupancy rate, the higher the constant returns to scale and Scale Efficiency. If there is no reason to consider capacity excess as valued traditional output, the occupancy rate may be related to hospital efficiency.

### Econometric analysis of the determinants of inefficiency

The selected Tobit model for explaining the observed hospital inefficiencies was earlier presented. The model contained the following variables: hospital size (SIZE), hospital ownership (OWN), proportion of outpatient department visits as a proportion of inpatient days (OPDID) and bed occupancy rate (BOR).

Table 5 presents the Tobit regression model results.

**Table 5:** Results of tobit model

Variable	Coefficient	t	*P* > |t|
SIZE	-0.3173112	-3.50	0.004**
OWN	0.919808	0.79	0.446
OPDID	-0.0950934	-2.47	0.028*
BOR	-1.097405	4.24	0.001**
cons	1.376723	4.67	0.000
Sigma	0.1448706		
	**p < 0.01;	*p < 0.05	
Observations Summary			
Number of observations			17
LR χ^2^			15.42
Prob > χ^2^			0.0039
Log likelihood			6.7537247
Pseudo R2			8.0570

The joint null hypothesis that H0 : β1 = β2 = β3 = β4 = β4 = 0 is rejected at the 1 % percent level of significance (Prob χ^2^ < 0.01). Therefore, we can conclude that the regression coefficients for the explanatory variables in our model are not equal to zero. It implies that our model as a whole fits significantly better than an empty model (i.e., a model with no predictors).

The coefficient for OPDIPD has a negative sign consistent with our a priori expectation, and statistically significant at the 5 percent level of significance.

A unit increase in the ratio of outpatient department visits to inpatient days would lead to a decrease in hospital expected inefficiency score by 0.0951, holding all other variables in the model constant. The higher a hospital’s OPDIPD, the lower the predicted inefficiency score.

The coefficient for Bed occupancy rate has a negative coefficient indicating that the lower the bed occupancy rate the higher the inefficiency score. A 1 % decrease in bed occupancy rate increases the inefficiency score by 1.097405 all other factors remaining constant. The score coefficient is statistically significant at the 1% level (*p* < 0.01).

Our results indicate that hospital size is a significant factor in explaining hospital inefficiency. The results indicate that the predicted inefficiency score for big hospitals is 0.32 points lower than for small hospitals all other factors remaining constant. This suggests that small hospitals are less efficient than big hospitals. The results also indicate that the predicted inefficiency score for government hospitals is 0.919808 higher than that for PNFP hospitals all other factors remaining constant, suggesting that PNFP hospitals are more efficient than government hospitals. However the coefficient for this variable is not significant.

### Scope for output increases/input reductions to improve efficiency

DEA calculates the efficiency score of a hospital of interest (target hospital) by comparing it with its efficient reference set. Thus, DEA outputs include an efficient reference set and corresponding weights (Refer to Table 4). Weighting of the efficiency reference set hospital inputs and outputs yields a hypothetical hospital (composite hospital) that produces as much or more than the target hospital but also uses fewer inputs. The difference between the inputs and outputs of the composite hospital and those of the target hospital indicates the input or output changes required to make the target hospital efficient.

Table 6 shows the input reductions and/or output increases that would have been required to make the individual pure technically inefficient hospitals efficient. To determine these, the inputs and outputs of a composite projected hospital are determined by multiplying the DEA- generated weights of each of the hospitals in the efficiency reference set used to identify an individual hospital’s inefficiency with each reference hospital’s actual outputs and inputs. The sum of the products of the multiplication is then compared to the inefficient hospital’s outputs and inputs.

**Table 6:** Efficiency scores and actual and target inputs and outputs quantities for inefficient hospitals according to VRS assumption

Hospital	Score-VRS	Input/output	Actual quantity	Target quantity	Difference	Percentage
Arua	0.855481	Medical Staff	198	164	-34	-17 %
		Beds	316	270	-46	-15 %
		OPD Visits	176689	176689	0	0 %
		Patient Days	90231	90,231	0	0 %
Fort Portal	0.921675	Medical Staff	189	174	-15	-8 %
		Beds	371	300	-71	-19 %
		OPD Visits	190665	190665	0	0 %
		Patient Days	88832	99260	10428	12 %
Jinja	0.86767	Medical Staff	219	190	-29	-13 %
		Beds	443	316	-127	-29 %
		OPD Visits	160387	160387	0	0 %
		Patient Days	108007	108007	0	0 %
Kabale	0.843276	Medical Staff	130	110	-20	-15 %
		Beds	252	203	-49	-19 %
		OPD Visits	138321	138,321	0	0 %
		Patient Days	70396	70,396	0	0 %
Soroti	0.941373	Medical Staff	178	168	-10	-6 %
		Beds	254	239	-15	-6 %
		OPD Visits	121629	121629	0	0 %
		Patient Days	96558	96558	0	0 %
Mubende	0.850297	Medical Staff	112	95	-17	-15 %
		Beds	175	149	-26	-15 %
		OPD Visits	86715	86715	0	0 %
		Patient Days	62456	62,456	0	0 %
Naguru	0.523887	Medical Staff	153	80	-73	-48 %
		Beds	100	52	-48	-48 %
		OPD Visits	35390	81,333	45943	130 %
		Patient Days	24382	24382	0	0 %
Rubaga	0.762422	Medical Staff	256	195	-61	-24 %
		Beds	271	207	-64	-24 %
		OPD Visits	155544	155544	0	0 %
		Patient Days	40380	61377	20997	52 %
Mbarara	0.976592	Medical Staff	197	192	-5	-3 %
		Beds	323	315	-8	-2 %
		OPD Visits	155185	155,185	0	0 %
		Patient Days	116277	116277	0	0 %

Naguru Hospital, for example, with a Technical efficiency score of 0.523887 (based on an efficiency reference set comprising Gulu with DEA generated weight of 0.408324; and Moroto with DEA generated weight of 0.077042) would need to increase its outpatient department visits by 45,943(130 %) in order to become technically efficient while maintaining its current level of inputs. Alternatively, it could also become technically efficient by reducing its total medical staff by 73 (48 %) and its bed capacity by 48 beds (48 %) as well, while maintaining the current level of outputs. The required changes per hospital vary; ranging from a required increase in OPD visits between 0 % and 130 % with an average of 14 %. For patient days, the required increases range from 0 to 52 % with an average of 7 %. For inputs, the required decreases for medical staff range from 3 to 48 % with an average of 17 % while for beds, the required decreases range from 2 to 48 % with an average of 20 %.

## Discussion

One approach to strengthening health services is through enhancing efficiency in utilization of existing resources to ensure that they are functioning to the best of their capacity with what is available so that the greatest health benefit possible may be attained. Improving efficiency is important at all levels, but the potential impact is particularly great in hospital services which use quite a substantial amount of health inputs. Measuring the efficiency of hospitals at the meso-level (e.g. regional) of the health system is useful for assessing and responding to variation in the performance of comparable hospitals. By directing attention to hospitals at the high and low extremes of efficiency, it is possible to gain insight into what makes production processes work in their setting and focus supportive efforts where they are most needed.

This study provides two main perspectives on hospital efficiency in Uganda. Firstly, the study reveals variations in the efficiency of the regional referral and large PNFP hospitals studied. The constant return to scale technical efficiency score varied from a minimum of 43.1 % and a maximum of 100 %; the pure technical efficiency score varied from a minimum of 52.4 % and a maximum of 100 % while the scale efficiency score varied from a minimum of 43.1 % to a maximum of 100 %. This indicates that despite facing similar resource-constrained conditions with similar inputs, some hospitals are more successful in converting inputs to outputs than others. The study identifies the following as determinants of hospital efficiency among the sampled hospitals: hospital size, bed occupancy rate and outpatient department visits as a proportion of inpatient days.

Secondly the study determines the level of output increases or input reductions that were required to be made by the inefficient hospitals in order to become relatively efficient. These changes would translate into a 45,943 increase in outpatient department visits and 45,943 in-patient days without changing the total number of inputs. In other words, these are the potential gains that should be reaped by the health sector at no extra cost if these inefficient hospitals were to operate efficiently. Alternatively, efficiency would be achieved by reducing the total number of medical staff by 216 and reducing the total number of beds by 454 without changing the total number of outputs. One can argue that these are idle resources that can be re-deployed to other levels of the health system. In the current situation of existing unmet need and low quality of care, these efficiency savings can be invested to expand access and/or improve quality of care.

However, our study indicates that small hospitals are less efficient than large hospitals suggesting that redeploying apparently excess hospital beds (thus reducing hospital size) is likely to further reduce hospital efficiency. Additionally, given the level of unmet need in Uganda for a number of hospital services like surgery (43); the small number of health worker inputs per hospital as compared to established staffing norms and the decision-making responsibilities accorded to the hospital managers, our results should guide efforts to increase utilization rather than to evaluate resource allocation. This is supported by our study finding that increasing bed occupancy rate reduces hospital inefficiency.

The assessment of the efficiency of regional and large PNFP hospitals in Uganda as undertaken in this study provides an important complement to existing knowledge about performance of these hospitals and demonstrates use of routinely available data (through the HMIS) to contribute to improving efficiency of the various hospitals. The findings indicate hospitals at the high and low extremes of efficiency and the input and output changes required to make the hospitals more efficient. The findings can be useful to managers in directing their efforts and to gain further insight into factors that facilitate and inhibit production processes in various hospital contexts.

Application of DEA to assess hospital efficiency in Africa has increased in recent years. Our study contributes to a growing literature on efficiency analysis in Africa generally and specifically to an incipient literature on hospital efficiency in Uganda. The last published DEA study in Uganda reports an average technical efficiency score of 97.3 % among the hospitals studied [28] which is higher than the average technical efficiency score of 91.4 % obtained in our study. Similar studies in Uganda’s regional peers Kenya [6] and Tanzania [44] found average technical efficiency scores of 95.6 % and 76.9 % respectively. While it is not possible to compare the true efficiency of hospitals across settings because the technical efficiency scores are calculated in relation to the frontier of efficiency in each sample, it is noteworthy that the average technical efficiency score of 91.4 % reported in our study is lower than that reported in Kenya but higher than that reported in the study in Tanzania. However, there were differences in the hospitals studied, with the Kenya study focusing on public hospital and the Tanzania study focusing on faith based hospitals (private not profit) while our study focused on both.

### Study limitations and suggestions for further research

The study reported in this paper has a number of limitations and also raises some areas for further study.

A key limitation of the study is the small sample size involved. This is important since DEA produces results that are very sensitive to measurement error, especially in small samples. A small sample size may result in many hospitals becoming efficient by default (as a result of not having a comparator from within the small sample)**.** To address this problem to some extent, we used the rule of thumb that the sample size should be at least three times the sum of number of inputs and outputs. Also, the study included 17 out of the 18 (94 %) regional referral hospitals active during the study period. The sample size for a similar study in Eritrea [4] comprised 19 out of 20 hospitals (95 %) this number is not very much different from the sample size in our study.

Data envelopment analysis (DEA) the analysis technique used in this paper is deterministic; meaning that estimation of an error term is not involved. The absence of an error component means that any deviation from the production possibilities frontier is attributed to inefficiency; there is no allowance for consideration of “random noise” such as epidemics, natural disasters which are out of a hospital’s direct control and yet may affect its performance. It may be interesting to conduct the same study using econometric techniques that take into consideration random errors (e.g. SFA) in order to validate the efficiency scores from this study. Use of both DEA and SFA has been recommended by Coelli and colleagues and implemented by Jacobs [45] and Linna [46].

Due to lack of data, the study did not include expenditure on pharmaceuticals and other non-pharmaceutical supplies among the inputs. Also, as one of the inputs the various categories of health workers (doctors, nurses, Clinical officers, Laboratory technicians and anesthetic officers) were all lumped together as medical staff due to absence of a detailed breakdown of staff categories for all the sampled hospitals. This may mask differences in the composition of the available health workers (e.g. doctors, nurses, lab technicians etc.) in the different hospitals considered. Additionally, even within the same health workforce category, the quality of labour input may vary depending on individual health worker skills, motivation and professional experience.

Estimation of allocative efficiency requires data on quantities of health service outputs, health system inputs, and input prices. Since we did not have the data on input prices, we focused only on the technical efficiency and scale efficiency of the hospitals in our study.

The hospitals included in the study were all referral hospitals. It is however possible that there were differences in the severity of cases treated in each hospital. This data was not available since the study relied on secondary data. Significant differences in the severity of cases treated could affect the number of cases hospitals dealt with relative to their staff numbers and bed numbers and could therefore have an impact on the results of the analysis. The hospitals treating a large number of severe cases, for example, may handle fewer cases, and will thus appear to be relatively inefficient. Adjusting for case-mix in the analysis would have helped address this problem and also provide better justification for the input variable reductions or output increases proposed for the hospitals identified by this study as being inefficient.

Whereas the data used for the study is relatively recent, some of the highlighted limitations above mean that the results of this analysis cannot be uncritically fed into current decision making. The study however illustrates the potential usefulness of efficiency analyses as conducted in this study using routinely available data. These analyses would be made useful if they are used to measure trends in efficiency and productivity of hospitals over time [6, 8, 17, 33] for example using Malmquist Total Factor Productivity Index analysis. This a potential area for further research that would entail collecting inputs and outputs data for a number of years and would permit tracking and comparison of hospital efficiency over time.

## Conclusion

In order to improve hospital performance, health policy makers need information about how well the hospitals are utilizing the resources they receive. This study has shown how DEA methods can be applied at the meso-level (regional) of the health system to gain insight into variation in efficiency across hospitals using routinely available data. The findings provided empirical evidence of the technical efficiency of the sampled hospitals and the input and output changes required to make the inefficient hospitals relatively efficient. The study also identifies hospital size (SIZE), bed occupancy rate (BOR) and outpatient visits as a proportion of inpatient days (OPDIPD) as the main driver of efficiency among hospitals. Further work is however required to support hospital managers in putting the results to use in enhancing efficiency. Hospitals identified at the high and low extremes of efficiency should be investigated further to determine how and why production processes are operating differently at these hospitals.

Given the small number of health worker inputs per hospital as compared to established staffing norms and limited control of hospital managers over inputs at the hospital level as well as the degree of unmet need for hospital services in Uganda, efforts to enhance efficiency should focus on strategies to increase demand and utilization of services (outputs) rather than reduction of inputs. As policy makers gain insight into mechanisms promoting hospital services utilization in hospitals with high efficiency, such as engagement with community leaders and improving quality of care, they can develop context-appropriate strategies for supporting hospitals with low efficiency to improve their service and thereby better address unmet needs for hospital services in Uganda.

## Abbreviations

CRS, constant returns to scale; DEA, data envelopment analysis; DMU, decision making unit; FY, financial year; HSSIP, health sector strategy and investment plan (HSSIP 2010/11-; MOH, Ministry of Health; NGO, Non-Governmental Organization; OPD, Out Patient Department; PNFP, private not for profit; SUOs, standard units of output; VRS, variable returns to scale
